# Relationship between the distribution of intra-retinal hyper-reflective foci and the progression of intermediate age-related macular degeneration

**DOI:** 10.1007/s00417-023-06180-4

**Published:** 2023-08-11

**Authors:** Aditya Verma, Giulia Corradetti, Ye He, Muneeswar G. Nittala, Marco Nassisi, Swetha B. Velaga, Jonathan L. Haines, Margaret A. Pericak-Vance, Dwight Stambolian, SriniVas R. Sadda

**Affiliations:** 1 Doheny Eye Institute, Pasadena, CA USA; 2 Department of Ophthalmology and Visual Sciences, University of Louisville Health Eye Specialists, Louisville, KY USA; 3 Department of Ophthalmology, David Geffen School of Medicine at the University of California, Los Angeles, Los Angeles, CA USA; 4 Tianjin Key Laboratory of Retinal Functions and Diseases, Tianjin Branch of National Clinical Research Center for Ocular Disease, Eye Institute and School of Optometry, Tianjin Medical University Eye Hospital, Tianjin, China; 5 Department of Clinical Sciences and Community Health, University of Milan, Milan, Italy; 6 Fondazione IRCCS Ca’ Granda Ospedale Maggiore Policlinico, Milan, Italy; 7 Department of Population & Quantitative Health Sciences and Cleveland Institute for Computational Biology, Case Western Reserve University, Cleveland, OH USA; 8 John P. Hussman Institute for Human Genomics, University of Miami Miller School of Medicine, Miami, FL 33136 USA; 9 Department of Ophthalmology, University of Pennsylvania, Perelman School of Medicine, Philadelphia, PA 19104 USA

**Keywords:** Intraretinal hyper-reflective foci, Intermediate age-related macular degeneration, Late age-related macular degeneration, Optical coherence tomography, Retinal pigment epithelium

## Abstract

**Purpose:**

To assess the relationship between the distribution of intra-retinal hyper-reflective foci (IHRF) on optical coherence tomography (OCT) and progression of intermediate age-related macular degeneration (iAMD) over 2 years.

**Methods:**

Cirrus OCT volumes of the macula of subjects enrolled in the Amish Eye Study with 2 years of follow-up were evaluated for the presence of iAMD and IHRF at baseline. The IHRF were counted in a series of 5 sequential en face slabs from outer to inner retina. The number of IHRF in each slab at baseline and the change in IHRF from baseline to year 2 were correlated with progression to late AMD at 2 years.

**Results:**

Among 120 eyes from 71 patients with iAMD, 52 eyes (43.3%) of 42 patients had evidence of both iAMD and IHRF at baseline. Twenty-three eyes (19.0%) showed progression to late AMD after 2 years. The total IHRF count increased from 243 at baseline to 604 at 2 years, with a significant increase in the IHRF number in each slab, except for the innermost slab 5 which had no IHRF at baseline or follow-up. The IHRF count increased from 121 to 340 in eyes that showed progression to late AMD. The presence of IHRF in the outermost retinal slabs 1 and 2 was independently associated with a significant risk of progression to late AMD. A greater increase in IHRF count over 2 years in these same slabs 1 and 2 was also associated with a higher risk of conversion to late AMD.

**Conclusions:**

The risk of progression to late AMD appears to be significantly associated with the distribution and extent of IHRF in the outermost retinal layers. This observation may point to significant pathophysiologic differences of IHRF in inner versus outer layers of the retina.


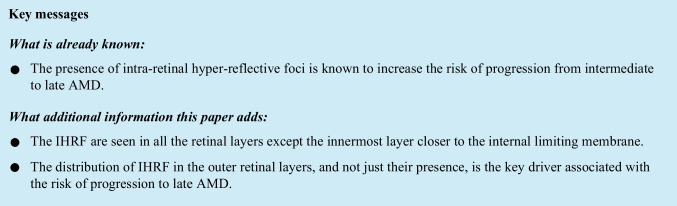


## Introduction

Intra-retinal hyper-reflective foci (IHRF) on optical coherence tomography (OCT) have been increasingly implicated as one of the most important predictors for progression to late-stage age-related macular degeneration (AMD), commonly defined as atrophy and/or macular neovascularization (MNV) [1–11]. First reported by Khanifer et al. in 2008 [12], these OCT biomarkers have been defined as discrete, > 3-pixel size, well-circumscribed hyper-reflective lesions within the neurosensory retina, having a reflectivity at least comparable to the retinal pigment epithelium (RPE) band [13, 14].

Migration of stressed RPE cells into the retina as well as inflammatory cells has been proposed as potential originating sources of these lesions [15]. Microglial cell proliferation and subsequent trans-retinal migration from the outer retina inwards during the course of inflammation in various disease processes including AMD have been proposed as a mechanism by which IHRF may arise [16, 17]. Other investigators have proposed RPE as a more plausible source of origin of these foci, especially those eyes that are destined to develop late AMD [18–20]. Previous reports have also shown that migration of RPE cells towards the deep capillary plexus (DCP) is a critical initial event in the development of intraretinal neovascularization (type 3 MNV) over time [19, 20]. Furthermore, a recent study by Cao et al. [21] conceptualized the origin of IHRF from “sloughed retinal pigment epithelial (RPE) cells” and proposed a progression sequence based on the available literature, OCT evidence, and immune-histochemical analysis of donor eyes [19–24].

Given the apparent prognostic and pathophysiologic importance of IHRF [4], understanding their prevalence, distribution, and evolution of these lesions over time would appear to be of importance. The Amish Eye Study provides a useful resource for the study of AMD biomarkers over time as multimodal imaging data was collected prospectively at baseline and 2 years using a standardized protocol, and the Amish represent a relatively homogenous population with regard to both environmental and genetic factors which minimizes confounding factors [25–27].

Thus, in this study, we analyze the extent and distribution of IHRF over 2 years in eyes with intermediate AMD (iAMD) [28, 29] from elderly Amish individuals, and evaluate the association of these IHRF parameters with the development of late AMD [30, 31].

## Methods

In this retrospective longitudinal, Institutional Review Board-approved (University of Pennsylvania, University of Miami, Case Western Reserve University, and University of California—Los Angeles) study, we analyzed en face spectral-domain OCT images at baseline and 24 months later. The study was performed in accordance with the Health Insurance Portability and Accountability Act and adhered to the tenets of the Declaration of Helsinki.

### Subject enrollment

Elderly subjects were recruited from three settlements in Ohio, Indiana, and Pennsylvania, as a part of Amish Eye Study. To be enrolled in the study, subjects had to have age ≥50 years, self-identify as being Amish, and be a member of a sibship in which at least one individual was reported to have AMD. The characteristics of this cohort have been described in previous reports [11, 25].

All subjects underwent volume OCT scans of both eyes using the Cirrus high-definition (HD)-OCT (Carl Zeiss Meditec, Dublin, CA; 512×128 macular cube; 6×6 mm scan region centered at the fovea) at baseline and at month 24. All images were de-identified and exported and sent to the Doheny Image Reading and Research Lab (Pasadena, CA, USA) for analysis by certified reading center graders.

Overall, OCT volume data from 120 eyes of 71 patients with iAMD were evaluated for the presence and distribution of IHRF in various retinal slabs (as described in the next section) at baseline and was followed up over a 2-year period. When analyzing IHRF, all eyes with evidence of retinal disease other than iAMD (evidence of late AMD, diabetic retinopathy, epiretinal membrane etc.), as well as eyes with gross segmentation errors which could impact retinal slab selection, were excluded from the final analysis. Late AMD was graded using the color fundus pictures and OCT scans [28, 30, 31].

### IHRF analysis protocol

**Fig. 1:**
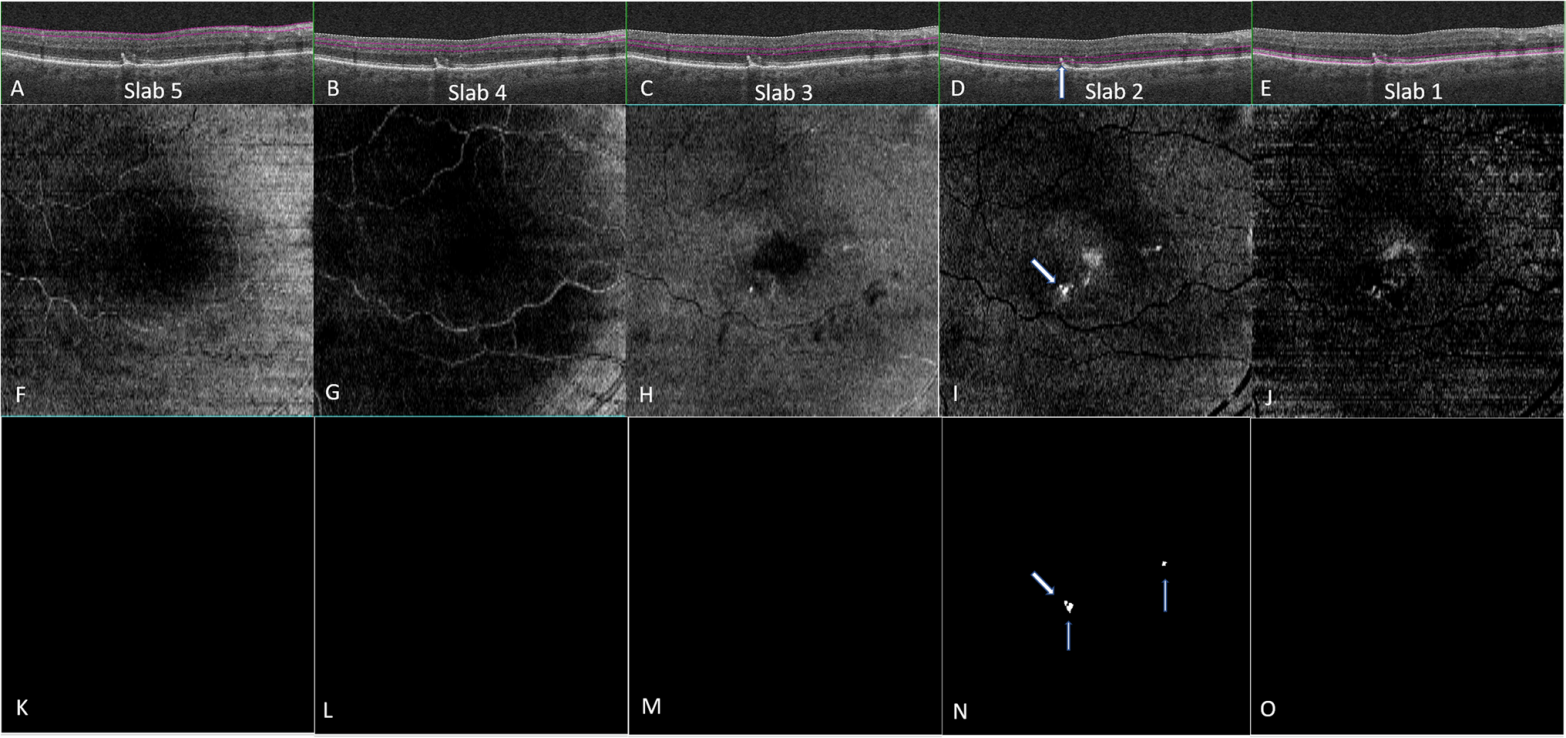
Quantification of intra-retinal hyper-reflective foci. **A**–**E** Five consecutive retinal slabs of equal thickness (one-fifth of the retinal thickness) were obtained between the internal limiting membrane (ILM) and the retinal pigment epithelium (RPE). **F**–**J** En face images generated from the corresponding 5 retinal slabs. IHRF appear as the brightest hyper-reflective features on the image (white arrows). **K**–**O** Following thresholding and binarization in ImageJ, the IHRF are isolated from the background and a total of 3 distinct lesions are identified, all present in slab 2 only (white arrows). Slab location: slab 1 (range: 80–100): External limiting membrane (ELM) till the outer border of interdigitation zone; slab 2 (range: 60–80): Inner border of outer nuclear layer (ONL) till inner border of ELM; slab 3 (range: 40–60): Mid-region of inner nuclear layer (INL) till the inner border of ONL; slab 4 (range: 20–40): Inner border of inner plexiform layer (IPL) till the middle border of INL; slab 5 (0–20): Nerve fiber layer till the inner border of IPL

A five-step process was used for the IHRF analysis:

1. Identification of the IHRF on structural OCT B-scans.
2. Generation of 5 en face slabs from the mid-retina.
3. Thresholding and binarization of slabs using ImageJ.
4. Manual removal of hyper-reflective artifacts surrounding the IHRF.
5. Quantification of IHRF (number) from each slab (Fig. 1).

#### Identification of IHRF on structural OCT B-scans

All structural OCT B-scans in the volume were reviewed for the presence of IHRF as described previously [5]. Briefly, IHRF were defined as discrete, well-circumscribed hyperreflective lesions at least 3 pixels in size, located within different layers of the neurosensory retina, and a reflectivity equal or more than that of the retinal pigment epithelial (RPE) band [21]. The assessment for presence of IHRF was performed by two independent masked graders (AV and YH), and a IHRF was deemed to be present if the grader had >90% confidence that it was present in at least one B-scan image, as per the reading center guidelines. In regions where there were clusters of IHRF, each IHRF was counted as a separate lesion as long as the individual foci were clearly separated by intervening normal reflectivity. Discrepancies between graders resolved by open adjudication or by the senior reading center investigator (SRS) for cases where the graders could not come to an agreement.

#### Generation of sequential en face retinal slabs

The Cirrus OCT provides automatic segmentation of the internal limiting membrane (ILM) and inner RPE surface and allows the user to adjust surface positions to generate custom slabs. In order to allow the frequency of IHRF at different levels of the retina to be quantified, a series of 5 sequential slabs each representing 20% of the retinal thickness were generated between the RPE and ILM, with the inner surface of the slab following the ILM contour and the outer surface following the RPE contour. Slab 1 was located just above the RPE (spanning roughly from the external limiting membrane (ELM) to the RPE), slab 2 spanned roughly from the inner border of the outer nuclear layer (ONL) to the ELM, slab 3 spanned roughly from the middle of the inner nuclear layer (INL) to the inner border of the ONL, slab 4 spanned roughly from the inner border of the IPL to the middle of the INL, and slab 5 was just below the ILM (spanning from roughly from the ILM to the inner border of the IPL).

#### Thresholding and binarization of extracted slabs

Before exporting the slabs, graders confirmed the precise location of IHRF on each slab by correlation with the B-scans, in order to differentiate IHRF from confounding sources of hyper-reflectivity such as focal regions of RPE elevation that were inadvertently included in the slab or blood vessels. The 5 slabs were exported from the OCT device and imported into ImageJ (version 1.50; National Institutes of Health, Bethesda, MD; available at http://rsb.info.nih.gov/ij/index.html) for further analysis [32]. In case the IHRF was located at the boundary between two slabs, it was counted as twice, once in each slab (as it appeared on the en face image).

As the en face ellipsoid zone (EZ) normalized reflectivity has been measured and reported previously [33], the same threshold was applied to each retinal slab to isolate the IHRF lesions (which given that they are defined to be as bright as the RPE band, exceeded this EZ reflectivity threshold). Graders also reconfirmed that the IHRF were not due to confounders (e.g., bright spots due to segmentation artifact or retinal blood vessels) by inspection of the corresponding B-scans. Threshold values were adjusted accordingly to try to further minimize these apparent artifactitious IHRF lesions without disappearance of true IHRF lesions. Any residual IHRF artifacts were manually removed, using the free hand selection tool in order to generate an artifact-free threshold-refined slab for binarization.

#### Quantification of IHRF [6, 7, 34]

After binarization of this threshold-refined slab, the number of IHRF in each slab was then calculated using the “particle analysis” tab. IHRF counts were obtained for each slab and for each eye with IHRF at baseline and at 24 months.

### Statistical analysis

All analyses were performed using statistical software (SPSS Statistics v 21.0; IBM Corp., New York, NY, USA). The numbers of IHRFs and the change in IHRF over 2 years were considered as continuous variables, whereas the conversion from iAMD to late AMD was considered as a nominal and dichotomous variable. The normalcy of distribution of data was confirmed using the Shapiro-Wilk test. Paired *t* test was used to analyze the difference in the IHRF count between baseline and 24-month follow-up. Univariate, followed by multivariate logistic regression was used to analyze the risk association of IHRF location (qualitative), and frequency and change (quantitative) in different retinal slabs as independent variables, on the development of late AMD at 2 years as the dependent variable to generate odd’s ratio. Inter-grader agreement [intraclass correlation coefficients (ICC)] for IHRF quantification between two independent graders (AV and YH) was assessed. *P* values less than or equal to 0.05 were considered statistically significant.

## Results

**Fig. 2:**
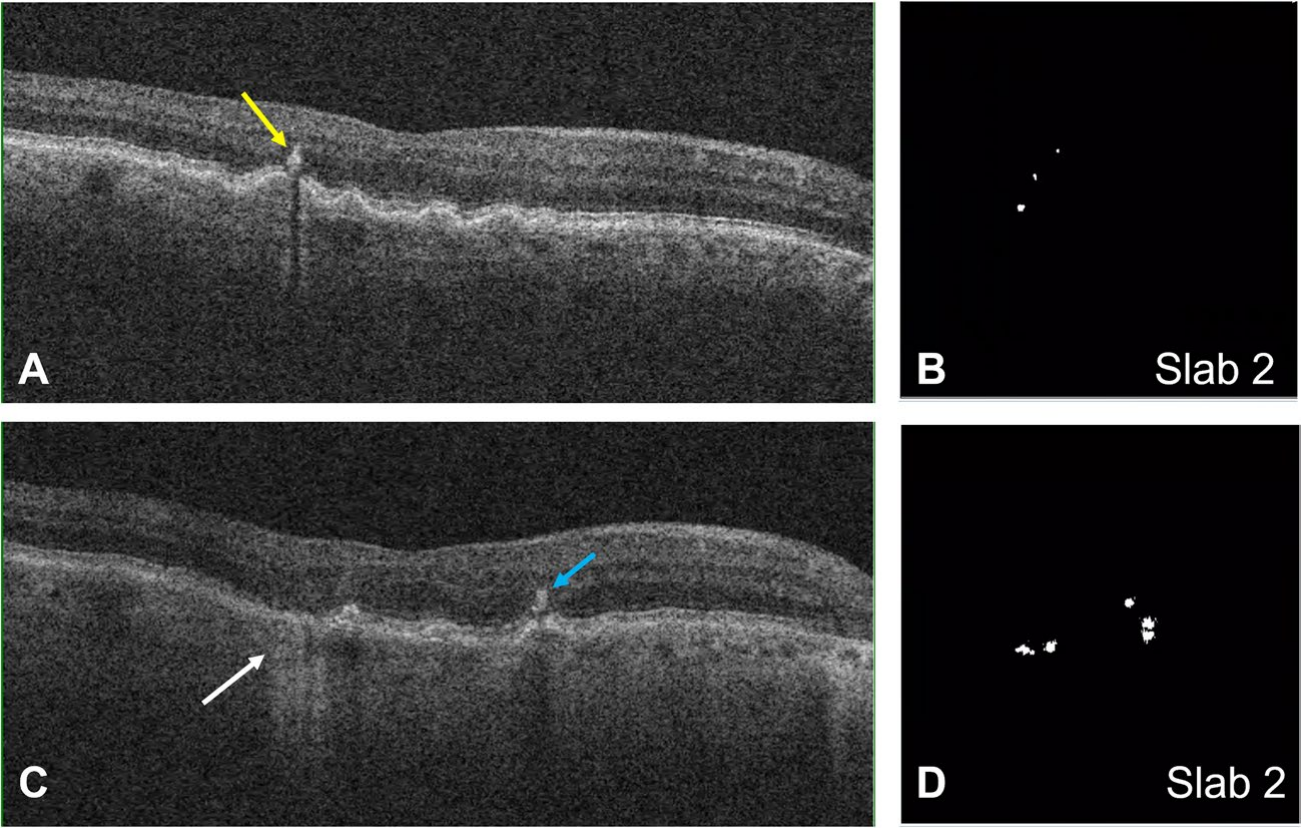
Structural optical coherence tomography (OCT) B-scan (**A**) of an eye with intermediate age-related macular degeneration at baseline showing multiple drusen and an intraretinal hyperreflective focus (IHRF; yellow arrow). The ImageJ-generated binarized en face image (**B**) from the outer retina highlights this lesion as well as two smaller lesions further superiorly. OCT B-scan (**C**) from the same region of the same eye at month 24. The large drusen at this location has collapsed with disappearance of the IHRF in this region and the appearance of hyper-transmission (white arrow) and loss overlying outer retinal bands indicative of atrophy. New IHRF (blue) arrows have appeared and are also evident as multiple foci in different locations on the corresponding ImageJ-generated binarized en face image (**D**) from the outer retina

The mean age ± standard deviation [SD] (range) of the 71 patients with iAMD at baseline was 72.27 ± 10.52 (51–97) years, 36 (50.7%) were female. Of these, 52 eyes (43.3%) of 42 patients had the evidence of both iAMD and IHRF, which were included in the final analysis [mean age ± SD (range) = 74.17 ± 9.37 (52–97) years, 24 were female]. IHRF were present bilaterally in 10 patients (20 eyes), and unilaterally in 32 patients (16 in the right eye and 16 in the left eye). Twenty-three eyes (19.0 %) showed progression to late AMD at the 2-year follow-up period. Figure 2 illustrates an example of a drusen with overlying IHRF which evolves to a region of atrophy by month 24. Additionally, 28 eyes with intermediate AMD which did not have IHRF at baseline developed IHRF over the 2-year period. Individuals who progressed were significantly older (76.30 ± 6.01 years), compared to the subjects who did not progress to late AMD (65.19 ± 10.14 years); *P* <0.001.

**Fig. 3:**
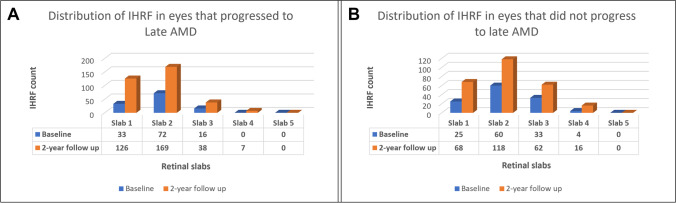
Bar-graph showing the distribution of IHRF in various retinal slabs at baseline and at 2 years in eyes that progressed to late AMD (**A**) and those that did not (**B**)

Quantitative analysis of this IHRF subgroup revealed a total of 243 IHRF lesions (average 4.6 per eye) at baseline, and 604 (11.6 per eye) [increase by ×2.48] at the 2-year follow-up. In those eyes that showed progression to late AMD, however, the IHRF count increased from 121 to 340 [increase by ×2.86]. Figure 3 shows the distribution of IHRF in various retinal slabs at baseline and at 2 years, in eyes that progressed to late AMD and in those that did not.

With regard to IHRF distribution, the IHRF count (average ± standard deviation, SD) at baseline by slab was as follows (Table 1): slab 1 = 58 (0.04 ± 0.35); slab 2 = 132 (0.1 ± 0.88); slab 3 = 49 (0.04 ± 0.49); slab 4 = 4 (0.0 ± 0.07); and slab 5 = 0. At the 2-year follow-up, the counts were as follows: slab 1 = 194 (0.14 ± 1.35); slab 2 = 287 (0.21 ± 1.77); slab 3 = 100 (0.07 ± 0.79); slab 4 = 23 (0.02 ± 0.26); and slab 5 = 0. There was a statistically significant increase in IHRF counts in slabs 1 (*P*=0.001), 2 (*P*=0.001), 3 (*P*<0.001), and 4 (*P*=0.02) over 2 years, though at both baseline and year 2, slab 4 had significantly fewer IHRF compared to outer slabs located closer to the RPE. The unweighted *k* value for intergrader repeatability was 0.90 (95% CI: 0.80–0.95; *P* <0.001) for IHRF quantification.

**Table 1:** Distribution of IHRF in various retinal slabs at baseline and at 2 years in the study cohort

Retinal slab	IHRF count at baseline *N* (average ± SD)	IHRF count at 2 years *N* (average ± SD)	*P* value
Slab 1	58 (0.04 ± 0.35)	194 (0.14 ± 1.35)	0.001
Slab 2	132 (0.1 ± 0.88)	287 (0.21 ± 1.77)	0.001
Slab 3	49 (0.04 ± 0.49)	100 (0.07 ± 0.79)	<0.001
Slab 4	4 (0.0 ± 0.07)	23 (0.02 ± 0.26)	0.02
Slab 5	0	0	NA

Slabs 1–5: Five consecutive structural B-scan OCT slabs, extending from the inner limiting membrane (inner border of slab 5) to the inner surface of the RPE (outer border of slab 1); each representing one-fifth of the retinal thickness; *IHRF*, intra-retinal hyper-reflective foci; *N*, count; *SD*, standard deviation

### Progression analysis

#### Risk of progression to late AMD with baseline quantitative IHRF distribution (Table 2)

The quantitative analysis (considering the distribution of IHRF in each slab, as continuous variables) showed that there was a significant risk of progression from iAMD to late AMD (OR = 1.21, 95% CI = 1.07–1.38, *P* = 0.003). When considering the distribution of IHRF lesions in each slab by univariate regression analysis, a significant association was noted between the presence of IHRF at baseline in slab 1 (*P*<0.001) and slab 2 (*P*=0.002) with the development of late AMD over 2 years. On multivariate analysis, only IHRF in slabs 1 (*P*=0.02) and 2 (*P*=0.04) were independently associated with the significant risk of progression to late AMD over 2 years.

**Table 2:** Association of the distribution (number) of IHRF at baseline with the development of late AMD at 2 years

Distribution of IHRF at baseline	Univariate			Multivariate		
	OR	95% CI	*P* value	OR	95% CI	*P* value
Slab 1	2.13	1.40–3.25	**<0.001**	1.74	1.10–2.75	**0.02**
Slab 2	1.46	1.14–1.86	**0.002**	1.38	1.02–1.87	**0.04**
Slab 3	1.11	0.88–1.40	0.36	1.13	0.67–1.92	0.65
Slab 4	NA	NA	NA	NA	NA	NA
Slab 5	NA	NA	NA	NA	NA	NA

Slabs 1–5: Five consecutive structural B-scan OCT slabs, extending from the inner limiting membrane (inner border of slab 5) to the inner surface of the RPE (outer border of slab 1); each representing one-fifth of the retinal thickness; *IHRF*, intra-retinal hyper-reflective foci (considered as continuous variables); *OR*, odd’s ratio; *CI*, 95% confidence interval; *NA*, unable to compute due to low (slab 4)/no (slab 5) sample size. The *P* values in bold are significant

#### Change in the IHRF number over 2 years and risk of progression to late AMD (Table 3)

There was a significant association between the increase in IHRF count over 2 years for slabs 1 (*P*=0.001) and slab 2 (*P*=0.001) and progression to late AMD at 2 years. Notably, the presence of IHRF in slab 4, the innermost slab with IHRF present, was not associated with progression. However, multivariate regression analysis revealed that IHRF increases in the same slabs, i.e., slab 1 (*P*=0.02) and slab 2 (*P*=0.05) were independent predictors of progression to late AMD.

**Table 3:** Association of the change in IHRF number over 2 years with progression to late AMD at 2 years

Change in IHRF from baseline to 2-year follow-up (Δ)	Univariate analysis			Multivariate analysis		
	OR	95% CI	*P* value	OR	95% CI	*P* value
Δ slab 1	1.46	1.18–1.80	**0.001**	1.34	1.05–1.70	**0.02**
Δ slab 2	1.25	1.09–1.42	**0.001**	1.25	1.00–1.57	**0.05**
Δ slab 3	1.21	0.95–1.54	0.12	0.77	0.49–1.22	0.27
Δ slab 4	1.27	0.78–2.08	0.33	1.12	0.59–2.12	0.72
Δ slab 5	NA	NA	NA	NA	NA	NA

Abbreviations: Slabs 1–5: Five consecutive structural B-scan OCT slabs, extending from the inner limiting membrane (inner border of slab 5) to the inner surface of the RPE (outer border of slab 1); each representing one-fifth of the retinal thickness; *IHRF*, intra-retinal hyper-reflective foci (considered as continuous variables); Δ, change in the number of IHRF from baseline to 2-year follow-up; *OR*, odd’s ratio; *CI*, 95% confidence interval; *P* values in bold are significant

#### Risk of progression to late AMD with baseline qualitative IHRF distribution (Table 4)

Univariate analysis showed that although the presence of IHRF (as categorical variables) in slabs 1 and 2 were equally significant risk factors for progression, multivariate analysis revealed that the presence of IHRF in slab 2 (*P*<0.001) carried a higher risk as compared to slab 1 (*P*=0.03).

**Table 4:** Association of presence of IHRF (*qualitative*) at baseline with the development of late AMD at 2 years

Presence of IHRF at baseline		Univariate			Multivariate		
		OR	95% CI	*P* value	OR	95% CI	*P* value
Slab 1	No	1			1		
	Yes	13.28	4.65–37.94	**<0.001**	4.18	1.18–14.48	**0.03**
Slab 2	No	1			1		
	Yes	29.59	7.84–109.21	**<0.001**	18.04	4.11–79.07	**<0.001**
Slab 3	No	1			1		
	Yes	3.07	0.98–9.58	**0.05**	0.69	0.16–2.90	0.61
Slab 4	No	NA	NA	NA	NA	NA	NA
	Yes	NA	NA	NA	NA	NA	NA
Slab 5	No	NA	NA	NA	NA	NA	NA
	Yes	NA	NA	NA	NA	NA	NA

Abbreviations: Slabs 1–5: Five consecutive structural B-scan OCT slabs, extending from the inner limiting membrane (inner border of slab 5) to the inner surface of the RPE (outer border of slab 1); each representing one-fifth of the retinal thickness; *IHRF*, intra-retinal hyper-reflective foci (considered as categorical variables); *OR*, odd’s ratio; *CI*, 95% confidence interval; *NA*, unable to compute due to low (slab 4)/no (slab 5) sample size. The *P* values in bold are significant

## Discussion

In this study, we evaluated the extent and distribution of IHRF over time and their impact on the risk for progression from intermediate AMD to late AMD over 2 years. We observed that IHRF are more extensive in the outer retinal layers compared to the inner retina and that IHRF more than double in number over a 2-year period. We also observed that that the risk of progression to late AMD over 2 years is significantly increased with the number of IHRF in slab 1 (OR = 2.13, 95% CI = 1.40–3.25; *P*=<0.001) and 2 (OR = 1.46, 95% CI = 1.14–1.86; *P*=0.002) spanning from roughly the inner border of ONL to the outer most portion of the neurosensory retina (Table 2). Also, the distribution of IHRF in each slab bears a significant risk of progression from iAMD to late AMD (OR = 1.21, 95% CI = 1.07–1.38, *P* = 0.003). Furthermore, IHRF distribution in the same slabs [slab 1 (OR = 1.74, 95% CI = 1.10–2.75; *P*=0.02) and 2 (OR = 1.38, 95% CI = 1.02–1.87; *P*=0.04)] appeared to be associated with the greatest risk and were the only independent predictors of progression. Similar trend of risk association was observed with the change in IHRF in each retinal slab over 2 years (Table 3). These findings highlight the fact that not only the presence but also the distribution of IHRF is important in terms of the risk for progression to late AMD.

The lack of an association between IHRF in one of the innermost slabs (slab 4) and progression to late AMD may be a finding of particular interest. On the one hand, if one assumes that these slab 4 IHRF are migrated RPE cells and further assumes that a greater distance between these foci and the RPE implies a greater duration of migration time, one might anticipate that such an eye may be more prone to late AMD since IHRF would have been present in the eye for a longer time [11, 21]. On the other hand, this finding may suggest that IHRF that are located in the inner retina may be distinct and of a different source of origin than IHRF in the outer retina (slabs 1–3) [19, 21–23]. For example, IHRF in the outer retinal layers may be more likely to be distressed RPE cells that have migrated into the retina [35, 36]. We have observed that the choriocapillaris is more severely impaired in regions in which there are IHRF and have hypothesized that this relative ischemia may trigger these cells to migrate into the retina to seek nourishment from the deep capillary plexus (DCP) of the retina [19, 37–39]. Furthermore, we have hypothesized that these RPE cells stimulate the DCP to produce type 3 MNV, which is typically preceded by the presence of IHRF [38, 39]. If the intended target of migrating RPE cells is the retinal DCP, there may not be significant drive for these cells to migrate further internally and may explain why the major risk for progression to advanced AMD is conferred by IHRF in the outer retina [19]. In contrast, IHRF located in the inner retina (slab 4 in our case) may be inflammatory cells, microglia, or other cells that have arrived from the retinal circulation rather than having come from the RPE layer [15–17]. Testing these hypotheses, however, will likely require prospective studies with higher-resolution novel imaging devices (e.g., adaptive optics combined with fluorescence lifetime imaging) which are not currently available. It is notable that IHRF were never observed in the innermost slab (slab 5) which is primarily composed of the ganglion cell layer and nerve fiber layer. It is uncertain what properties of these innermost layers preclude the development of migration of IHRF to this location.

Although the number of IHRF in the inner retina at baseline was not a significant predictor of progression, an increase in IHRF over 2 years was associated with higher risk for progression to late AMD over 2 years on univariate analysis. While this was not an independent predictor on multivariate analysis, this does suggest that increasing IHRF in the inner retina may still be a biomarker of disease progression. It should be noted that increasing IHRF in the outer retina was also associated with progression to late AMD, and an increase in the outer most slabs (slabs 1 and 2) was both independent risk factors for progression.

Similar to previous studies, our eye level analysis indicated that the presence of any IHRF was associated with a higher risk for progression to late AMD [7, 11, 13, 21]. Because of this increased risk, subjects with intermediate AMD and IHRF have been suggested as potential candidates for early intervention trials of potential dry AMD therapies. Recently, post hoc analyses have shown that both pegcetocoplan (FDA approved) and avacincaptad can reduce the conversion from incomplete RPE and outer retinal atrophy (iRORA) to complete (cRORA) [40]. Thus, there has been interest in determining whether treatment could slow eyes with IHRF from progressing to iRORA and/or cRORA [40–43]. The rate of these conversions within 2 years, however, may be relatively low, which may make these biomarkers unfeasible for use as clinical trial endpoints. Of note, our study demonstrated that IHRF can dramatically increase in number over a 2-year period. As increasing numbers of IHRF are associated with a greater risk for progression, preventing this increase with potential therapeutic agents may provide an alternative and more clinically feasible endpoint.

Our study has some limitations which must be considered when assessing our findings. First, although the OCT data was collected as part of prospective cohort study, the analysis in this report was not pre-specified. Second, while we quantified the number of IHRF, other morphometric parameters such as the shape and size of these lesions were not considered. Third, IHRF were quantified in somewhat arbitrary slabs that were based on a percentage of the retinal thickness and not in specific retinal layers. Although we considered segmenting the various retinal layers and quantifying the IHRF in each layer, the thickness of the layers varies throughout the macula, and important characteristics like reflectivity of certain layers like Henle’s layer may be impacted by underlying pathology such as drusen. This can decrease the confidence of graders to assess the accuracy of segmentation and further IHRF analysis. Furthermore, the advantage of only requiring segmentation of the RPE and ILM boundaries is that these structures can be evaluated more reliably, and segmentation errors can be readily identified and corrected or excluded. Fourth, while the Amish population offers some advantages with regard to relatively uniform genetic and environmental (e.g., no smoking, similar diet) background, our observations in the Amish may not necessarily extrapolate to the general population. Finally, although we counted a large number of IHRF, the number of eyes was relatively small. Our study also has several strengths including a standardized OCT protocol and the use of certified graders with demonstrated high grading reproducibility.

In summary, the extent, distribution, and progression of IHRF appear to be associated with the risk for progression to late AMD. Once IHRF are apparent, their numbers can dramatically increase, even over a 2-year period. IHRF are most prevalent in the outer retinal layers and these outer IHRF seem to be associated with the greatest risk for progression to late AMD. The cellular origin of IHRF at different levels of the retina warrants further study.

## Declarations

### Disclaimer

Although the co-author (Vas Sadda) is an editor of the journal, there was no involvement with the peer-review process for this article.
